# AAV8 vector induced gliosis following neuronal transgene expression

**DOI:** 10.3389/fnins.2024.1287228

**Published:** 2024-02-29

**Authors:** Faye McLeod, Elaine McDermott, Shermin Mak, Darren Walsh, Mark Turnbull, Fiona E. N. LeBeau, Andrew Jackson, Andrew J. Trevelyan, Gavin J. Clowry

**Affiliations:** Centre for Transformative Neuroscience, Newcastle University Biosciences Institute, Newcastle upon Tyne, United Kingdom

**Keywords:** gene therapy, AAV vector, epilepsy, neuroinflammation, opsins, microglia, astrocytes

## Abstract

**Introduction:**

Expression of light sensitive ion channels by selected neurons has been achieved by viral mediated transduction with gene constructs, but for this to have therapeutic uses, for instance in treating epilepsy, any adverse effects of viral infection on the cerebral cortex needs to be evaluated. Here, we assessed the impact of adeno-associated virus 8 (AAV8) carrying DNA code for a soma targeting light activated chloride channel/FusionRed (FR) construct under the CKIIa promoter.

**Methods:**

Viral constructs were harvested from transfected HEK293 cells *in vitro* and purified. To test functionality of the opsin, cultured rodent neurons were transduced and the light response of transduced neurons was assayed using whole-cell patch-clamp recordings. *In vivo* expression was confirmed by immunofluorescence for FR. Unilateral intracranial injections of the viral construct were made into the mouse neocortex and non-invasive fluorescence imaging of FR expression made over 1–4 weeks post-injection using an IVIS Spectrum system. Sections were also prepared from injected mouse cortex for immunofluorescence staining of FR, alongside glial and neuronal marker proteins.

**Results:**

*In vitro*, cortical neurons were successfully transduced, showing appropriate physiological responses to light stimulation. Following injections *in vivo*, transduction was progressively established around a focal injection site over a 4-week period with spread of transduction proportional to the concentration of virus introduced. Elevated GFAP immunoreactivity, a marker for reactive astrocytes, was detected near injection sites associated with, and proportional to, local FR expression. Similarly, we observed reactive microglia around FR expressing cells. However, we found that the numbers of NeuN+ neurons were conserved close to the injection site, indicating that there was little or no neuronal loss. In control mice, injected with saline only, astrocytosis and microgliosis was limited to the immediate vicinity of the injection site. Injections of opsin negative viral constructs resulted in comparable levels of astrocytic reaction as seen with opsin positive constructs.

**Discussion:**

We conclude that introduction of an AAV8 vector transducing expression of a transgene under a neuron specific promotor evokes a mild inflammatory reaction in cortical tissue without causing extensive short-term neuronal loss. The expression of an opsin in addition to a fluorescent protein does not significantly increase neuroinflammation.

## Introduction

Optogenetics is a powerful and flexible tool for temporally and spatially precise manipulation of neuronal activity in a cell-class specific manner, leading to the idea that it could be used to treat various neurological conditions. Traditional closed-loop neuronal control has used electrical stimulation, but this activates neurons indiscriminately, and furthermore, has to be delivered discontinuously, to avoid amplification of the electrical stimulation artifact. In contrast, optogenetics (Boyden et al., 2005; Deisseroth, 2015) allows continuous, rapid closed-loop neuronal control through activation or inhibition of neurons without stimulation artifacts (Zaaimi et al., 2023). A critical pre-requisite for optogenetic control systems, however, is to achieve persistent and stable expression of the opsins, while minimizing any transduction-related pathological response.

Adeno-associated virus (AAV) vectors have been highly effective in clinical gene therapy trials as they have a broad tropism, are easily synthesized, have low immunogenicity, and are non-pathogenic (Shirley et al., 2020). AAV vectors infect cells by attaching to primary receptors or co-receptors on the cell surface and being endocytosed. They are then transported to the nucleus where the virus is uncoated, and the inserted genome is released. The single-stranded DNA is then converted to double stranded DNA, before being transcribed and translated to express the transgene (Li and Samulski, 2020). Importantly, unlike lentiviral vectors, it does not integrate its DNA load into the host chromosome (Li and Samulski, 2020; Shirley et al., 2020).

As the AAV vector has no viral coding sequences, it elicits only a weak inflammatory or T1 interferon (IFN) response, although AAV particulates can degrade after exiting the endosome, leading to the formation of inflammatory complexes that can cause chronic damage (Costantini et al., 2000; Nayak and Herzog, 2010). Studies have reported no induction of gliosis in the brain and retina in response AAVs (Keiser et al., 2013; Giers et al., 2017) or even a reduction in the inflammatory response when AAVs have been used to deliver anti-inflammatory therapies in disease models (e.g., Xu et al., 2019; Xiang et al., 2022). Nevertheless, a neuroinflammatory response to the presence of viruses in the central nervous system (CNS) is predicted (Hordeaux et al., 2018; Perez et al., 2020). Such a response involves changes to both astrocytes and microglia (Liu et al., 2011; Liddelow et al., 2017; Giovannoni and Quintana, 2020; Nosi et al., 2021). Astrocyte activation is a prominent marker of neuroinflammation, resulting in proliferation, and growth and thickening of processes coinciding with upregulation of the astrocytic marker glial fibrillary acidic protein (GFAP; Sofroniew, 2009). This can also result in pro-inflammatory cytokine release (Colombo and Farina, 2016) contributing to neuronal damage and subsequent death. Microglia are the resident macrophages of the CNS, displaying differential morphology dependent on their activation state. They are the primary innate responder, reacting to cellular breakdown products and pathogens, producing cytokines and chemokines (Lau and Yu, 2001; Hanisch and Kettenmann, 2007; Sousa et al., 2017).

The purpose of this study was to gauge the extent of any immune responses induced by an AAV vectors delivering therapeutic expression of an opsin; our findings, however, may have general relevance to any form of gene therapy in the brain involving expression of transgenes delivered by AAV viral vectors. Having established appropriate opsin functionality *in vitro*, different doses of viral vector were injected into the sensorimotor cortex of mice. The extent of expression was measured by immunohistochemistry for a transgene marker protein. At the same cortical locations, we also co-labeled for either the reactive astrocytic marker GFAP, microglial marker IBA1, or neuronal marker NeuN. To control for inflammatory effects of the opsin, as opposed to the virus, we further examined the response to saline injections, and injections of a viral vector carrying a transgene that is expressed only in the cytosol, and not the membrane-bound opsin.

## Materials and methods

### Animals

All animal handling and experimentation was carried out under appropriate licenses issued by the UK Home Office under the Animals (Scientific Procedures) Act 1986 and approved by the Animal Welfare and Ethical Review Board of Newcastle University. Rats (3 pregnant animals) and mice (20 in total) were housed under a 12-h light, 12-h dark light cycle with free access to food and water.

### Viral vectors

Viral vectors were produced in-house for AAV8-CKIIa-stGtACR2-FusionRed (6.1 × 10^12^ vg/ml) as follows. HEK 293 cells were transfected with purified helper (E4, E2a and VA), AAV8 Rep/Cap and transfer plasmids containing the pyramidal cell specific CKIIa promoter, stGtACR2 (soma targeting *Guillardiatheta* anion-conducting channelrhodopsins) and FusionRed DNA using a polyethylenimine method (Boussif et al., 1995). Subsequently, cells were manually harvested, centrifuged and pelleted. Crude viral particles in the supernatant were then removed. The virus was column purified using an AKTA (GE Life Sciences) chromatography system and concentrated using Amicon ultracentrifuge filters (Millipore). Viral titer was measured by real-time PCR using serial dilutions then aliquoted and frozen at −80°C. Control experiments were also conducted using another viral vector, AAV8-CKIIa-GFP (8 × 10^12^ vg/ml), purchased from UNC Vector Core (aliquoted and stored at −80°C). All procedures involving viral vectors were done under Newcastle University safety guidelines and approved by the Microbiological Hazards and Genetic Modification Safety Advisory Sub-Committee.

### Rat cortical cultures

Primary cortical cultures were prepared from embryonic day 18 embryos of Sprague–Dawley rats. In brief, cortices were isolated from 6 to 8 embryos and stored in dissection media (1 x Hanks balanced salt solution supplemented with 1 mM HEPES and 10 mM glucose) on ice. Collected tissue was transferred to a conical tube and the dissection media was removed. The tissue was digested twice using papain solution (10 units/ml) for 20 min by incubation at 37°C, and then washed twice with the dissection medium and twice with the culture media containing Neurobasal media supplemented with 1x B27, 0.5% Fetal bovine serum (FBS), 1 mM L-glutamine and 0.5x antibiotic/antimycotic solution. Cells were dissociated within the culture media by trituration. A small sample of cell suspension was subsequently diluted and counted. Cells were plated at a 1 × 10^5^ cells/ml concentration in a 24-well plate containing sterile coverslips that have been previously poly-L-lysine coated overnight at room temperature (RT). Cells were maintained at 37°C, 5% CO₂ in ambient O₂ and 90% humidity. Culture media was changed 24 h post-plating, and thereafter, a half-media change, with culture media lacking FBS, was performed every 3 days. At 7 days *in vitro* (DIV) diluted viral vectors were added to the cells. At 14 DIV, cells were either fixed in 4% paraformaldehyde (PFA)/4% sucrose in phosphate buffered saline (PBS) for 20 min at RT prior to immunofluorescence staining or transferred to an electrophysiology rig, for recording tests of opsin functionality.

### Whole-cell patch clamp recording

Rat cortical cultures were transferred into the recording chamber of an upright Leica DMLFSA fluorescent microscope fitted with Micro Control Instruments micromanipulators and continuously perfused at 34°C with standard, oxygenated ACSF containing (in mM): 126 NaCl, 3 KCl, 1.25 NaH₂PO₄, 24 NaHCO₃, 10 Glucose, 1.2 CaCl₂ and 1 MgSO₄. Cells were voltage-clamped (at zero mV) in whole cell configuration using borosilicate glass patch electrodes (5–8 MΩ) filled with an intracellular solution containing (in mM): 125 K-methyl-SO4, 10 HEPES, 2.5 Mg-ATP, 6 NaCl; 290 mOsM and pH 7.35. Cells expressing the fluorescent reporter FusionRed (FR) were identified and functional opsin expression was confirmed by a reliable photo-induced current elicited by microscope fluorescence illumination. All data were collected using an Axopatch 200B amplifier, filtered (1 kHz) and digitized (10 kHz). Data was monitored online and analyzed offline using WinEDR and WinWCP software (freeware^1^). After recordings, all cultures were fixed as before.

### Intracranial injection of viral vectors and *in vivo* imaging

Using sterile procedures, animals were injected intraperitoneally with Buprenorphine (0.1 mg/kg) and Meloxicam (5 mg/kg) for pre-operative pain relief. Anesthesia was induced with 5% Isoflurane and maintained with 2–1.5% Isoflurane. A 4 mm diameter craniotomy was performed above the left cerebral hemisphere and viral vectors injected at two different sites in along the rostro-caudal axis of the sensorimotor cortex. Approximately, 1.5 μL of the viral vector suspension was injected per site at 3 depths; 0.8 mm, 0.5 mm, 0.2 mm at a rate of 50 nL/s. One dilution of the virus was injected at each site (undiluted stock, 1:10 or 1:100) using a 34 G beveled needle with a Nanofil syringe (World Precision Instruments). Following the final injection, a 4 mm glass coverslip was implanted. Finally, postoperative pain relief was applied, and the animal closely monitored for 4 weeks.

Non-invasive fluorescent *in vivo* imaging of mice was performed on an IVIS Spectrum system (Spectral Instruments Imaging systems) at 1, 3, and 4 weeks post-injection to track the progression of transduction. Animals were anaesthetized, transiently, with 5% isoflurane and maintained with 1.5% isoflurane, to allow stable imaging of the cranial window at 570 nm excitation, and fluorescence collected above 620 nm. Fluorescence was quantified as the emission radiance per incident excitation power (average radiant efficiency).

### Immunofluorescence

Mice were terminally anesthetized with an intraperitoneal injection of Ketamine (75 mg/kg) and Medetomidine (1 mg/kg). Mouse brains were removed following transcardial perfusion with buffered 4% PFA and placed overnight in the same fixative. All brains were placed in 30% sucrose in phosphate buffer solution (PBS) prior to sectioning. The tissue was frozen to −20°C and 40 μm coronal sections cut using a freezing microtome.

Sections were immunostained free-floating, and rat cortical cultures were stained directly on the glass coverslips. All sections underwent an antigen retrieval step by incubation in sodium citrate buffer (10 mM, pH 6.0, 100°C, for 10 min) and then washed with PBS. Samples were then placed in blocking solution (0.1% Triton X-100 and 5% donkey serum in PBS) for 2 h at RT, prior to incubation in primary antibody (see Table 1) diluted in blocking solution overnight at 4°C. Samples were then incubated with fluorophore-bound secondary antibodies raised in donkey (Life Technologies; 1:500 dilution) according to standard protocols and counterstained with DAPI (1:5000, Thermo Fisher Scientific) applied for 20 min at RT, then mounted and coverslipped with Fluoromount-G mounting media (SouthernBiotech).

**Table 1:** Primary antibodies used.

**Antibody to**	**Primary species**	**Dilution**	**Supplier**	**RRID number**
mCherry	Rabbit	1:500	Abcam	AB_2571870
NeuN	Mouse	1:1000	Abcam, Cambridge, UK	AB_10711040
GFAP	Chicken	1:1000	Abcam	AB_304558
IBA1	Goat	1:500	Abcam	AB_2224402
GFP	Rabbit	1:1000	Abcam	AB_303395

### Image acquisition and analysis

All images were acquired using an inverted Zeiss LSM 800 Airyscan confocal microscope or an upright epifluorescence Nikon Eclipse NiE microscope. Infected regions (FR positive areas of the neocortex) on the left hemisphere were identified using a 10x objective. Subsequently, all fluorescent images were taken with a 20x objective (numerical aperture = 0.5), image stacks acquired with a z-step of 2 μm and image resolution of at least 1,024 × 1,024 pixels at least 200 μm from the injection site. Images were also taken contralateral to the injection site, at homotypic locations. A minimum of three images were acquired from each section/hemisphere. For quantification purposes, the acquisition and analysis settings were kept constant for all images.

Image analysis was performed using FIJI software^2^. All settings remained constant for each marker of interest. Each GFAP or IBA1 positive profile was co-localized with the nuclear stain DAPI to confirm its cellular nature. Total area immunostained (μm^2^), percentage area immunostained (total area normalized to the image size), and neuronal counts, were achieved using thresholding techniques to segment the regions of interest for analysis. To test for these microglial morphological changes, we thresholded the images so that processes were eliminated and the % area occupied by IBA1 immunoreactive cell bodies was measured. For each stack of images, the z-series was flattened to produce a maximum projected 2D image. Pre-processing was performed by adjusting the brightness and contrast, or subtracting the background, to make the fluorescent areas easily distinguishable. Areas of interest were then outlined by adjusting the threshold to create a binary image. Subsequently, various filters were applied (e.g., ‘unsharp,’ ‘despeckle,’ ‘Otsu’) before the final analysis. The masked outline of each region was created to confirm that the parameters chosen were optimal.

### Statistical analyses

All data was taken from at least three independent experiments (unless otherwise stated in the figure legend). Statistical analyses were performed on GraphPrism 9. For some comparisons, we sought to examine various metrics of immunological response with respect to the local expression level, and so treated each section as an independent replicate and used linear regression analysis to compare transgene expression with markers of neuronal and glial responses. In reactive gliosis experiments we also combined data from individual animals (which included two injection sites with differing viral titres). We assessed data normality using Kolmogorov–Smirnov tests. Parametric (paired students *t*-test) were used where appropriate for comparing the injected and contralateral hemispheres in the same section. Statistical significance was accepted as *p < 0.05, **p < 0.01, and ***p < 0.001, and non-significant differences were indicated as ‘ns.’

**Figure 1:**
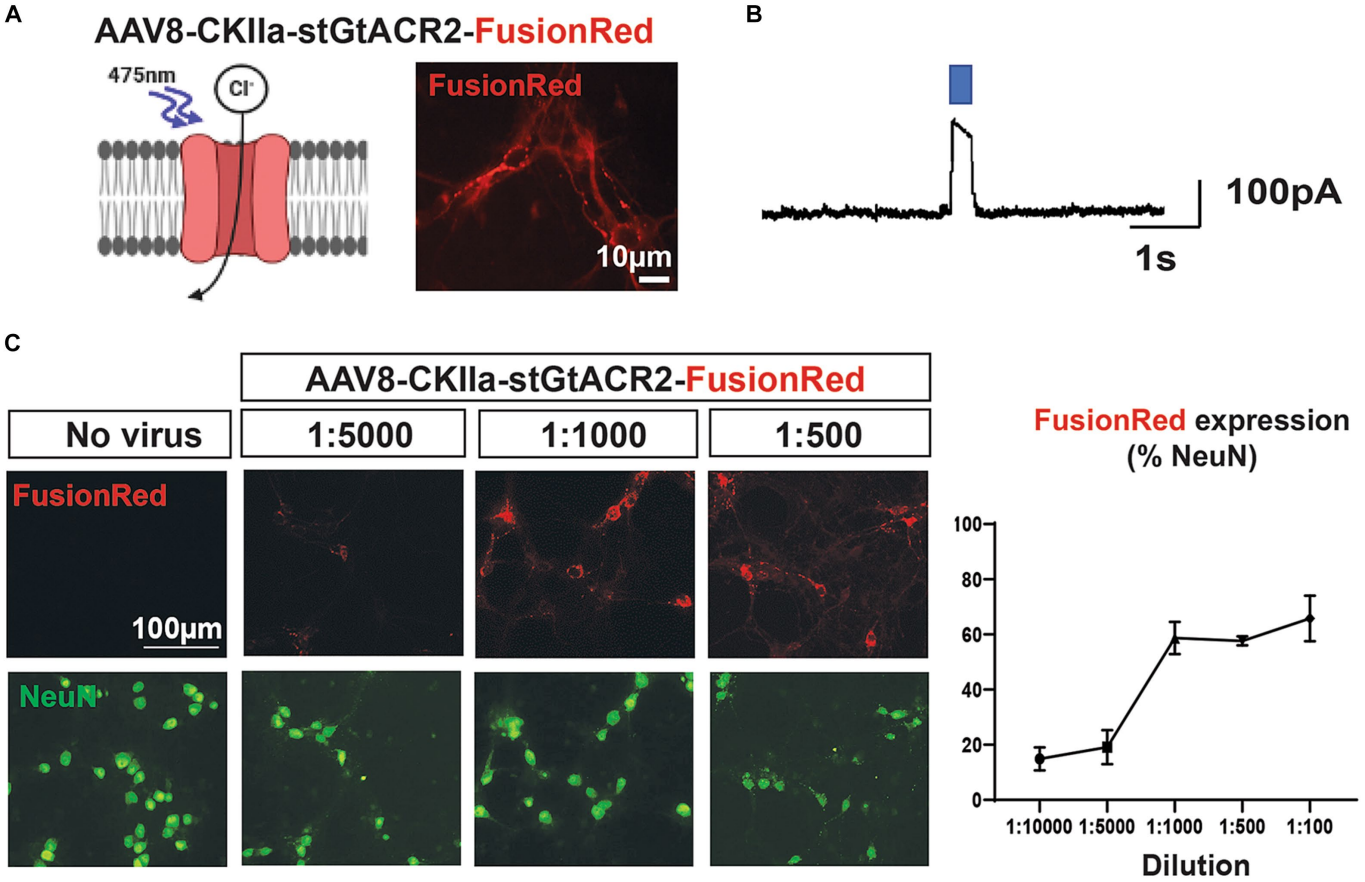
Dose-dependent transgene expression of AAV vector *in vitro*. **(A)** Left: the inhibitory opsin stGtACR2 responds to exposure to blue light (~475 nm), the light gated chloride channel opens resulting in cell hyperpolarization. Right: expression of AAV8-CKIIa-stGtACR2-FusionRed (1:1000 dilution) at 14 DIV in rat primary cortical cultures. Transduction was specific to pyramidal neurons (CKIIa promoter) and detected by the FusionRed fluorescent tag. **(B)** Whole-cell voltage clamp recording displaying functional light responses for the AAV8- CKIIa-stGtACR2-FusionRed viral vector. **(C)** Transduction profile of different AAV8-CKIIa-stGtACR2-FusionRed dilutions in cortical cultures (14 DIV). Cells treated with no virus (saline) were used as a comparative control. FusionRed in red, and neurons (NeuN) in green % +/-SEM of NeuN+ cells co-expressing FusionRed at each dilution. The schematic was produced with the aid of BioRender.com.

## Results

### The AAV8-CKIIa-stGtACR2-FR construct successfully transduced cortical neurons

We first examined whether rodent cortical neurons could be transduced with our inhibitory opsin/FR construct, using dissociated neuronal cultures derived from rat embryos, after 14 days *in vitro*. We made whole-cell patch clamp recordings of neurons, observing prominent currents, induced by illumination with blue light, indicative of opening of light-sensitive chloride channels (Figures 1A,B). At lower viral titres (1:5000-1:10000), less than 20% of neurons (cells immunopositive for NeuN) were immunopositive for FR; this rose to ~60% of cells being NeuN+/FR+ for dilutions between 1:1000 and 1:100 (Figure 1C). Virus negative cells did not display FR immunoreactivity or an optogenetic response.

**Figure 2:**
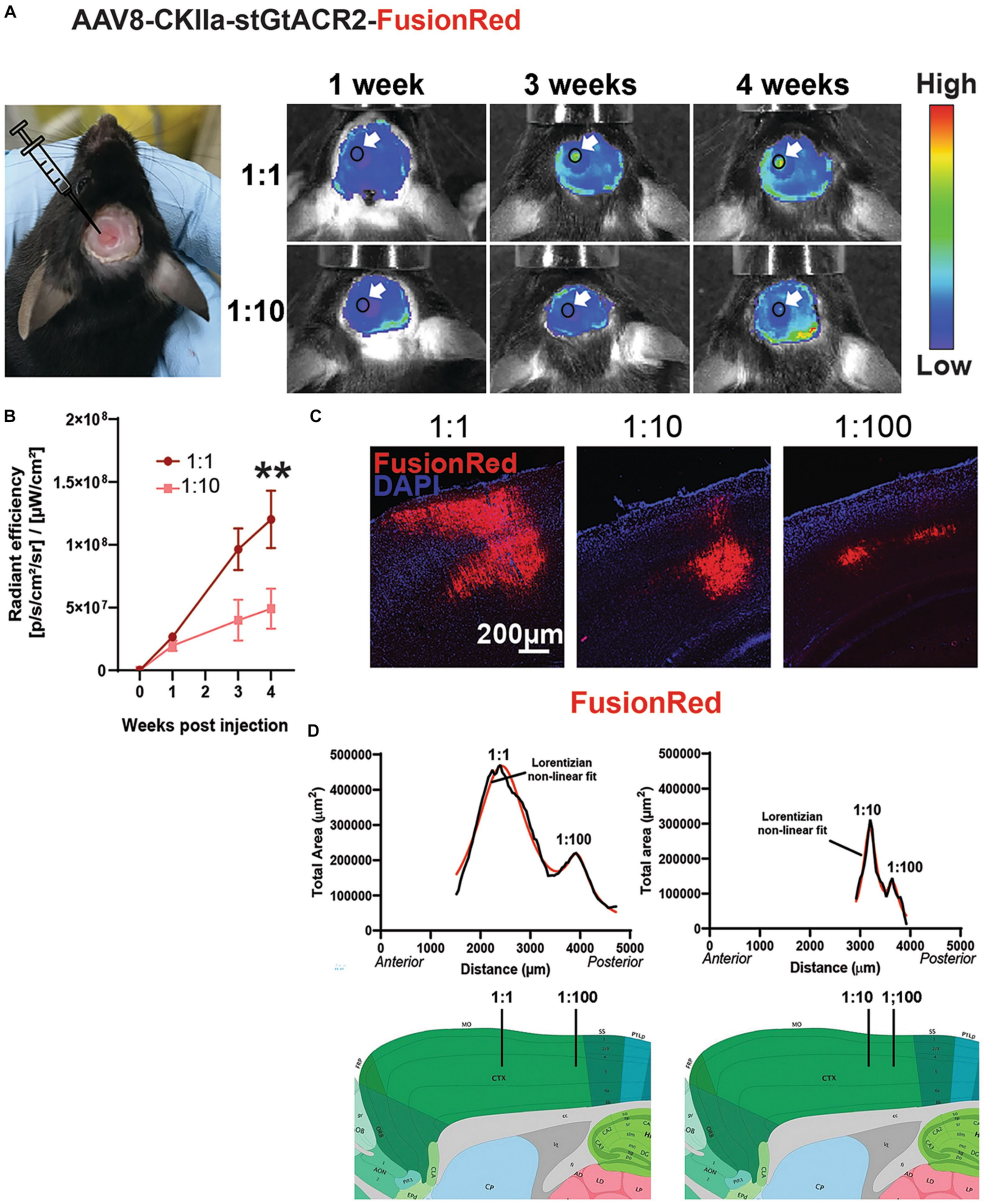
Transgene expression *in vivo*. **(A)** Left: example mouse with a cranial window above the left hemisphere following stereotaxic injections of two viral vector doses. Right: representative *in vivo* images showing change in fluorescence emission following expression of AAV8-CKIIa-stGtACR2-FusionRed (1:1 and 1:10 dilution) over 4 weeks. Arrows point to location of transgene expression. Autofluorescence from acrylic used to keep the coverslip in place can also be seen at the margins of the craniotomy. **(B)** Average radiant efficiency at each time point and dose, **p < 0.01, Data expressed as +/- SEM. **(C)** FR immunofluorescence (red) counterstained with DAPI (blue) after injection of different concentrations of AAV8-CKIIa-stGtACR2-FusionRed. **(D)** Quantification of transgene expression in the sensorimotor cortex; graph shows total cross-sectional area immunostained for each section from anterior to posterior cortex (μm^2^) in 2 mice [left = undiluted (1:1) and 1:100 dilution; right = 1:10 and 1:100 dilution] 4 weeks after intracranial injection. Each graph is fitted with a sum of two Lorentzian non-linear fit models (solid red line; *R*^2^ values = 0.96 for both animals) peaks are the predicted sites of injection marked with black lines on representative sagittal sections taken from the Allen mouse brain atlas (mouse.brain-map.org).

We achieved *in vivo* expression of the construct by multiple injections of the virus at different depths along a single injection track into the mouse cerebral cortex (see methods). Expression was monitored by imaging the FR fluorescence through a cranial window using the IVIS spectrum imaging system, showing a progressive increase over 4 weeks (Figures 2A,B). This occurred faster and to three times the fluorescence intensity when the virus was injected undiluted (1:1) compared to a 1:50 dilution (*n* = 3 mice/viral dose; *p* < 0.01 by Friedman test with Dunn’s *post hoc* test and data expressed as mean ± SEM). After 4 weeks, transduced animals were culled and the brains sectioned. FR immunoreactivity was imaged at low magnification in consecutive sections from exactly the same location in the injected and contralateral hemispheres. The cross-sectional area occupied by FR immunoreactive cells and processes was measured. Two injections of differing concentrations of virus were made in each ipsilateral hemisphere at different locations along the rostro-caudal axis. As can be seen in Figures 2C,D, the extent of spread of expression in both anterior/posterior and medial/lateral directions was approximately proportional to the concentration of virus injected, but each concentration successfully transduced neurons.

**Figure 3:**
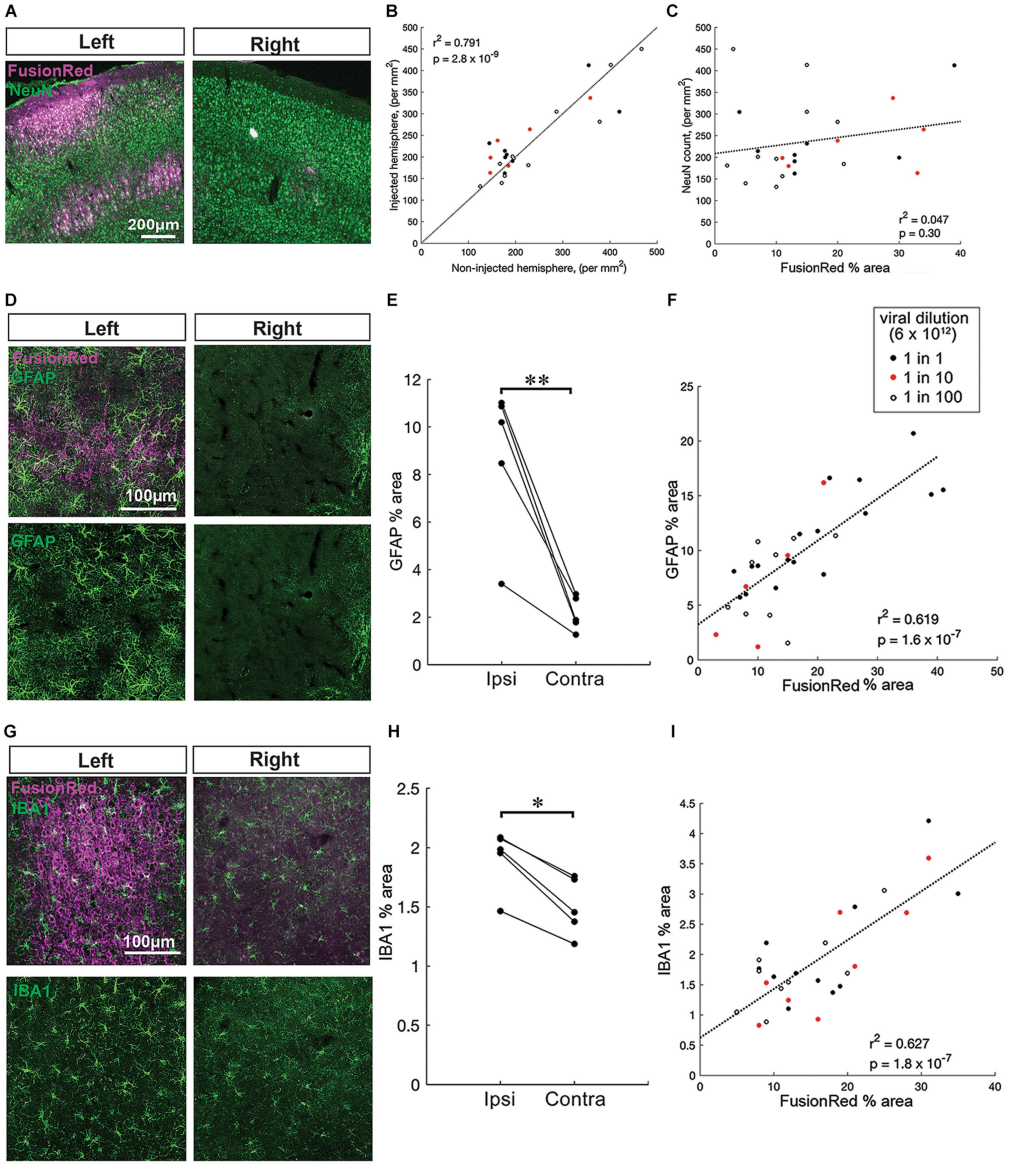
Pronounced chronic gliosis with higher AAV vector titres *in vivo*. **(A)** Representative images of NeuN+ neurons (green) co-labeled with FusionRed (magenta) near to an injection site in the left hemisphere and a corresponding site in the uninjected right hemisphere (no virus, control) 4 weeks after intracranial injection of AAV8-CKIIa-stGtACR2-FR into the sensorimotor cortex. **(B)** Positive correlation of NeuN+ cell count on the injected hemisphere compared to the non-injected hemisphere (*n* = 25 sections from 5 animals, *r*^2^ = 0.791, *p* = 2.8 × 10^−9^). **(C)** NeuN counts on the injected side showed no correlation with the local FusionRed expression (*n* = 25 sections from 5 animals, *r*^2^ = 0.047, *p* = 0.30). Viral dilution information: 1:1 = black, red = 1:10 and 1:100 = open white circles. **(D)** Top: zoomed in images of GFAP+ cells (green) with FusionRed (magenta) from the sensorimotor cortex of infected cortices (left) and non-infected cortices (right) 4 weeks after intracranial injection of undiluted AAV8-CKIIa-stGtACR2-FR. Bottom: the same GFAP+ images clearly displaying a substantial increase in GFAP+ astrocyte area occupied by GFAP+ immunoreactivity. **(E)** Higher immunoreactivity (GFAP % area) in the ipsilateral injected, hemisphere compared with the contralateral non-injected, hemisphere (*n* = 5 animals, **p < 0.01). **(F)** Significant correlation of GFAP % area with FusionRed % area (*n* = 31 brain sections from 5 animals, *r*^2^ = 0.619, *p* = 1.6 × 10^−7^). **(G)** Top: representative images of microglia (IBA1+; green) surrounding FR+ cells 4 weeks following an undiluted dose of virus injected into the left hemisphere alongside right hemisphere. Bottom: the same IBA1+ images. **(H)** Higher immunoreactivity (IBA1% cell bodies area) in the ipsilateral injected, hemisphere compared with the contralateral non-injected, hemisphere (*n* = 5 animals, *p < 0.05). **(I)** Highly significant positive correlation of IBA1% cell bodies area with FusionRed % area (*n* = 30 brain sections from 5 animals, *r*^2^ = 0.627, *p* = 1.8 × 10^−7^) that the schematic in **(A)** was produced with software tools available at https://www.biorender.com/.

### Higher AAV vector titers associated with chronic gliosis *in vivo*

In addition to immunostaining for FR, sections were double labeled for either NeuN, GFAP or IBA1 to assess neuronal numbers, astrocytosis or microgliosis, respectively. Within the injected hemispheres, the local expression of the transgene differed greatly depending on how close the brain section was to an injection site, and the dilution of viral vector used at that site (Figures 2C,D). To examine the neuronal and glial response to viral load at a more granular level, we therefore compared it with FusionRed labeling, which is a marker of the local viral load.

We first examined how NeuN counts differed in the injected hemisphere versus the non-injected hemisphere. The number of NeuN+ cells in the injected hemisphere were highly correlated with those in the opposite hemisphere at the same rostro-caudal level (Figures 3A,B; *n* = 25 brain sections, *r*^2^ = 0.791, *p* < 0.001 by linear regression). Notably, when the fit was constrained to pass through the origin, the gradient of the best fit was 0.98, with 95% confidence intervals between 0.91–1.06, meaning that the line of equivalence fell well within these intervals. We subsequently examined how NeuN counts varied with respect to the local viral expression, relative to the non-injected hemisphere (Figure 3C). NeuN counts on the injected side showed no correlation with the local FusionRed expression (Figure 3C; *n* = 25 brain sections, *r*^2^ = 0.047, *p* = 0.30 by linear regression). This suggests that transduction with the virus did not incur any significant neuronal loss within 4 weeks of injection.

Next, we assessed gliosis responses. Reactive astrocytes increase expression of GFAP and tend to extend their processes (Sofroniew, 2009), whereas reactive microglia tend to withdraw processes and their cell bodies increase in size (Davis et al., 2017). In every animal (*n* = 5), immunoreactivity for both GFAP (Figures 3D,E) and IBA1 (Figures 1G,H), was higher in the injected, compared with the non-injected, hemisphere (*p* < 0.001 for GFAP mean % area and *p* < 0.05 for IBA1 mean % cell bodies area by paired students t-test). We next compared the level of GFAP and IBA1 immunoreactivity, in each brain slice with FusionRed labeling (Figures 3F,I). Notably, both GFAP+ area and IBA1+ cell bodies area, in individual coronal sections, showed highly significant correlations with the level of local FusionRed expression in the same slice (GFAP, *n* = 31 brain sections, *r*^2^ = 0.619, *p* < 0.001, *p* < 0.001 by linear regression; IBA1, *n* = 30 brain sections, *r*^2^ = 0.627, *p* < 0.001 by linear regression). Collectively, this data suggests that local reactive astrocytic and microglial responses are dependent on the transduction of the AAV viral vector within 4 weeks of injection.

### Divergent time-course of transgene expression and astrocytosis

**Figure 4:**
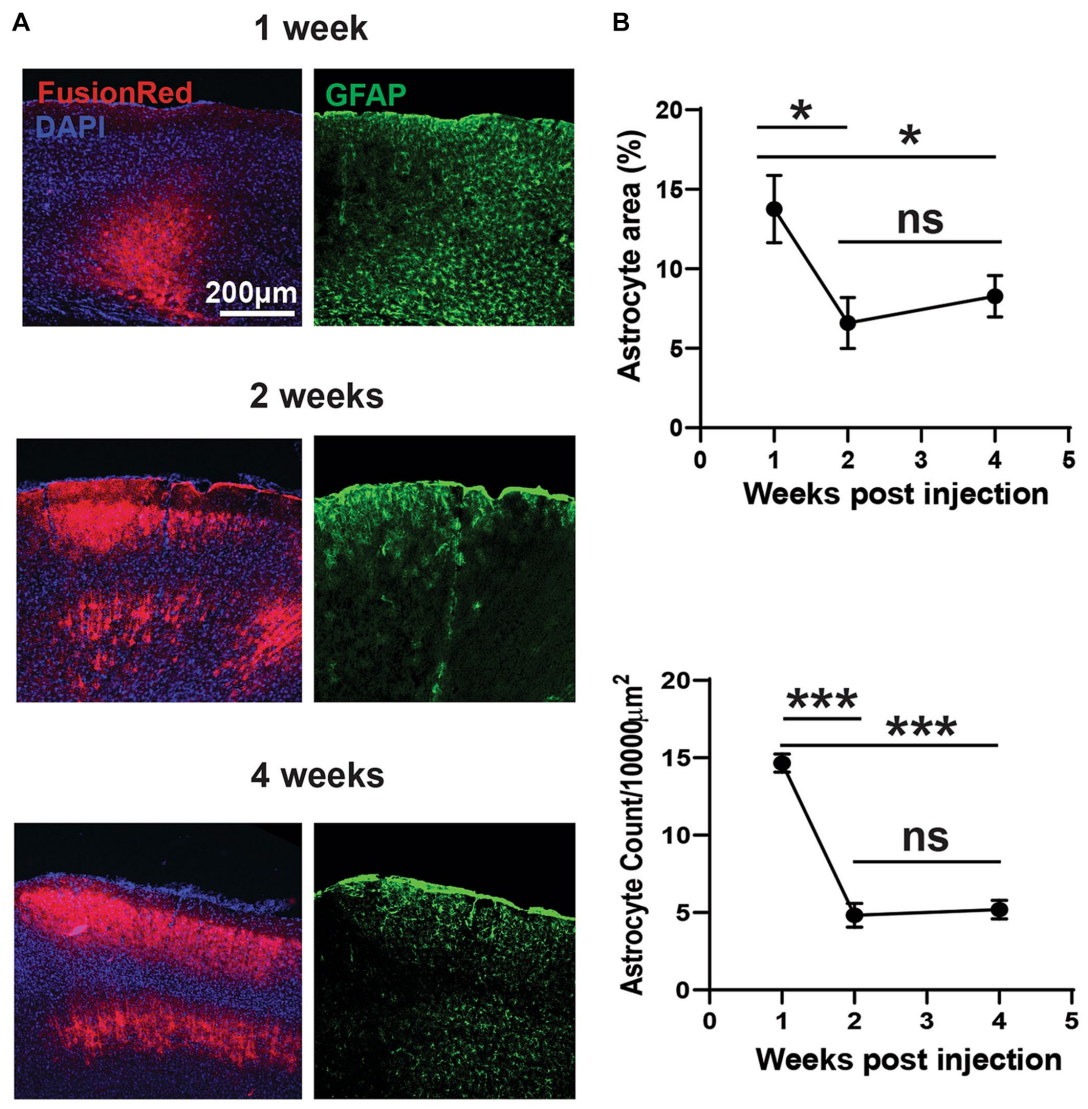
Divergent acute and chronic gliosis in response to AAV vectors. **(A)** Representative micrographs of AAV8-CKIIa-stGtACR2-FusionRed (undiluted stock) transduction (counterstained with DAPI) and reactive astrocyte (GFAP+; green) responses from 1 to 4 weeks post intracranial injection. GFAP+ immunostaining was acutely elevated at 1 week but decreased by 2–4 weeks (chronic). **(B)** Top: analysis of GFAP+ percentage area confirms this observation (*p < 0.05). Bottom: reduced reactive astrocyte count with time (***p < 0.001; *n* = 2–3 mice with 7–11 sections/time point). Data expressed as mean ± SEM.

Consistent with our *in vivo* data, *post mortem* analysis of FR immunofluorescence also showed evidence of increasing expression levels, particularly between 1 and 2 weeks post viral injection (undiluted). However, changes in GFAP immunoreactivity in the injected hemisphere followed a different time course and was significantly higher after 1 week post viral injection, but was reduced by approximately 50% by 2 weeks, with no further change at 4 weeks (Figures 4A,B; *p* < 0.05 for cell count, *p* < 0.001 for area measurements by one-way ANOVA with Tukey’s multiple comparison test; *n* = 2–3 mice with 7–11 sections/time point). GFAP expression remained elevated at 4 weeks, compared to the contralateral hemisphere. Measurements of the area occupied by FR and GFAP immunoreactivity in individual sections were shown to be closely and positively correlated 4 weeks post-injection (Figure 3F). These findings could be considered contradictory; greater early astrocytosis suggests that astrocytes primarily react to the presence of virus, not expression of the transgene. On the other hand, the spatial correlation of astrocytosis with the presence of FR expression suggests that chronic astrocytosis is a response to expression of the transgene products.

**Figure 5:**
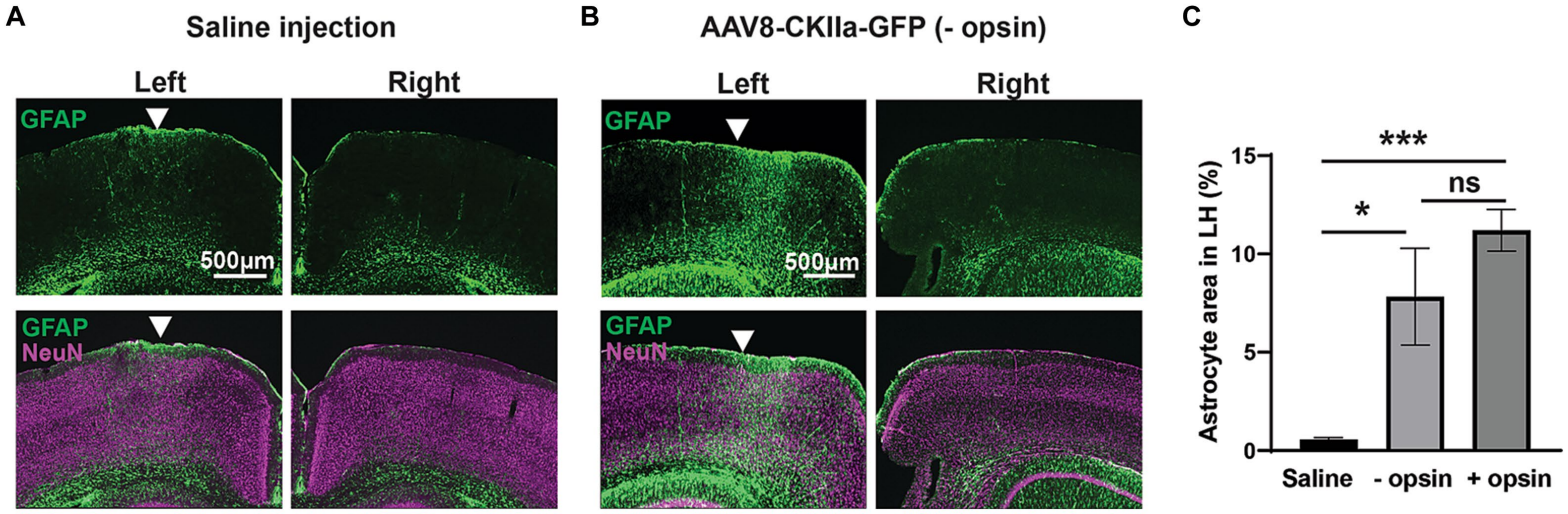
Gliosis in control experiments. **(A)** GFAP+ immunoreactivity (green) counterstained with the neuronal marker NeuN (magenta) following injection of saline into the left hemisphere (LH) with comparative right hemisphere. **(B)** The same staining following injection of AAV8-CKIIa-GFP in the LH and non-infected right hemisphere control. Note the marked increase in GFAP+ reactive astrocyte staining in the LH. **(C)** Quantification of GFAP+ area in the LH after saline, AAV8-CKIIa-GFP (opsin–) and AAV8-CKIIa-stGtACR2-FusionRed (opsin+) injected animals. Marked elevation in GFAP immunoreactivity for both viral vectors with and without opsins in comparison to saline injected animals (*p < 0.05 and ***p < 0.001; *n* = 1–3 mice with 6–17 sections for each). Data expressed as mean ± SEM.

To explore this further, we performed two different control experiments in order to differentiate between the various potential causes of the glial response. Firstly, saline without virus was injected into the cortex and shown to produce a minimal astrocytic response confined to close to the injection site (Figure 5A). This confirmed that the more extensive pattern of astrocytosis seen with the viral vector injections is largely attributable to the vector and/or its transgene, and less to the physical trauma of the injection. Secondly, we made injections of a viral vector carrying a transgene for the expression of a fluorescent reporter protein (GFP), without any opsin. Although expression of fluorescent proteins such as GFP can elicit an adaptive immune response, this only occurs when the protein is transduced in antigen presenting cells in the brain, primarily glial cells (Ciesielska et al., 2013) and not when the transgene is expressed intracellularly, as is the case here, where GFP was expressed under the control of a neuron specific promoter. Injection of a viral vector carrying a GFP+ opsin- transgene caused marked astrocytosis, significantly greater than with injection of saline alone and similar to a viral vector carrying a transgene with an opsin (Figures 5B,C; one-way ANOVA with Tukey’s multiple comparison test; *n* = 1–3 mice with 6–17 sections for each). From experiments described in Figures 3–5, we conclude that astrocytes respond vigorously to the presence of a viral vector, but this response subsides as the viral vector is cleared from the tissue, without disappearing completely. Expression of the transgene may contribute to maintaining astrocytosis. However, even though a transmembrane protein is more likely to interact with astrocytes than fluorescent reporter proteins located internally in the neuron, removal of opsin from the transgene does not contribute significantly to a reduction in astrocytosis. Within the context of developing a therapeutic application, the finding that opsin expression does not increase neuroinflammation may be considered a positive outcome.

## Discussion

Our study confirms that neuronal expression of a transgene carrying the stGtACR2 gene (a potentially therapeutic protein) can be achieved in the neocortex using viral vectors as previously shown (Kirchgessner et al., 2021). Functional chloride gating by the opsin was evident as a hyperpolarizing current induced by blue light, in patch clamp recordings of neurons *in vitro*. However, introduction of the viral vector and/or neuronal transduction *in vivo* induced astroglial and microglial reactivity suggestive of a marked inflammatory reaction. Reassuringly, however, this reaction did not lead to significant neuronal loss, at least within timeframe examined (4 weeks). While we did not examine longer periods, it seems likely that the peak risk to neurons would occur in the immediate aftermath of the viral injection, suggesting that this approach to gene delivery does not carry any substantial risk to neurons. However, we did not attempt to detect active neuronal death in our animals, for instance by using fluorojade labeling or activated caspase 3 immunostaining (Wang et al., 2008) which might have revealed small numbers of degenerating neurons.

It is known that viral vector infection of cerebral tissue leads to an inflammatory response (Thomas et al., 2001) and that an increase in GFAP expression is a reliable marker for inflammation (Zhang et al., 2017). For instance, lentivirus infection has been demonstrated to produce a dose dependent increase in astrocytic GFAP expression (Faideau et al., 2010). Increased GFAP expression also occurs with neurodegeneration in Alzheimer’s disease and multiple sclerosis (Abdelhak et al., 2018; Zhang et al., 2019). However, reactive astrocytes can be both pro- and anti- inflammatory in their actions (Zamanian et al., 2012) depending on whether activation occurs via the NRK-beta signaling (pro-inflammatory and leading to neurodegeneration; Lian et al., 2015) or JAK2-STAT3 signaling (scar forming, anti- inflammatory: Bush et al., 1999; Anderson et al., 2016). The failure to see significant neuronal loss, at least in the short term, indicates that astrocytosis does not necessarily lead to neurodegeneration, at least in our experimental paradigm.

It is widely assumed that astrocytic activation is dependent upon microglial activation (Liu et al., 2011; Jha et al., 2019) although evidence also points to the opposite occurring (Kwon et al., 2017). In any case, there is growing evidence for a dynamic cross talk between the two cell types, and with neurons, in the control of the inflammatory response (Kwon et al., 2015; Jha et al., 2019; Nosi et al., 2021). Here we detected small but significant changes in microglial morphology indicating adoption of a reactive phenotype. The changes appeared less marked than those observed in astrocytes. As with astrocytes, activated microglia can adopt a pro- or anti-inflammatory phenotype (Tang and Le, 2016). One role of microglia is to phagocytose viral particles following infection (Chhatbar and Prinz, 2021), as would be expected under our experimental conditions. However, phagocytosis of apparently healthy neurons can occur under inflammatory conditions (Fricker et al., 2012a; Neher et al., 2013) and neurons under stress, perhaps from expression of foreign proteins such as opsin/GFP constructs, can lead to the aberrant presentation of proteins and phospholipids on the neuronal surface, triggering phagocytosis (Wang and Neumann, 2010; Fricker et al., 2012b).

Although neuronal numbers appear to be preserved following viral vector injections, the function of neuronal networks may still be disrupted by the injection of the viral vector or expression of the transgene. For instance, reactive glia lose, or reduce, their capacity for playing a role in synaptic transmission. Reactive astrocytes in various disease states show impaired K+ buffering capacity (Tong et al., 2014; Kinboshi et al., 2020) glutamate re-uptake (Pardo et al., 2006; Matos et al., 2008; Green et al., 2021) and energetic support to neurons (Chao et al., 2019). Furthermore, reactive microglia may have an altered ability to monitor the functional states of synapses and prevent excess depolarization (Wake et al., 2009; Kato et al., 2016). On the other hand, promisingly, a recent study overexpressing a potassium channel transgene packaged into an AAV9 viral vector under control of the same CKIIa promoter found a robust reduction in seizure frequency in a mouse model of frontal lobe focal cortical dysplasia without any major behavioral effects (Almacellas Barbanoj et al., 2024). This controlled modification in network excitability, with minimal off target effects, is encouraging for AAV mediated transgene expression approaches.

In conclusion, we provide evidence that both astrocytes and microglia detect and react to an AAV vector and transgene introduced into the cerebral cortex resulting in chronic gliosis. In contrast, we found no evidence of neurodegeneration within a 4 week period. It is worth noting, however, that although use of AAVrh8 vectors to deliver gene therapy for Sandhof’s disease was shown to be safe in mouse and feline models (Cachon-Gonzalez et al., 2006; Rockwell et al., 2015) monkeys receiving a bilateral infusion in the thalamus and infusion in the left lateral ventricle developed necrotic thalamic lesions alongside ataxia, dyskinesias and loss of dexterity. This most likely resulted from overexpression of the transgene rather than the presence of the viral vector (Golebiowski et al., 2017). Further studies should investigate longer-term neurodegeneration, test viral vectors in other species and examine more subtle effects upon neuronal network activity as part of any effort to develop AAV mediated transgene expression as a gene therapy.

## Data availability statement

The raw data supporting the conclusions of this article will be made available by the authors, without undue reservation.

## Ethics statement

Ethical approval was not required for the studies on humans in accordance with the local legislation and institutional requirements because only commercially available established cell lines were used. The animal study was approved by Newcastle University Animal Welfare and Ethics Review Board and the UK Government Home Office. The study was conducted in accordance with the local legislation and institutional requirements.

## Author contributions

FM: Conceptualization, Methodology, Supervision, Writing – original draft, Writing – review & editing, Data curation, Formal analysis, Investigation. EM: Data curation, Investigation, Writing – review & editing. SM: Data curation, Investigation, Writing – review & editing. DW: Investigation, Writing – review & editing, Methodology. MT: Investigation, Methodology, Writing – review & editing. FL: Writing – review & editing, Supervision, Funding acquisition. AJ: Writing – review & editing, Conceptualization, Funding acquisition, Project administration. AT: Conceptualization, Formal analysis, Funding acquisition, Project administration, Writing – review & editing. GC: Conceptualization, Project administration, Writing – review & editing, Methodology, Supervision, Writing – original draft.

## Footnotes

^1^ http://spider.science.strath.ac.uk/sipbs/software_ses.htm

^2^ https://imagej.nih.gov/ij/download.html
